# Influence of antiplatelet medication and anticoagulation therapy after dental extractions on hospitalization: a retrospective 10-year study

**DOI:** 10.1186/s12903-024-05275-6

**Published:** 2024-12-13

**Authors:** Marie Sophie Katz, Rajae Benidamou, Mark Ooms, Marius Heitzer, Anna Bock, Dirk Elvers, Timm Steiner, Florian Peters, Frank Hölzle, Ali Modabber

**Affiliations:** Department of Oral and Maxillofacial Surgery, University Hospital RWTH Aachen, Pauwelsstraße 30, 52074 Aachen, Germany

**Keywords:** Postoperative bleeding, Antiplatelet therapy, Oral anticoagulation, Dental extraction, Hospitalization, Risk patients

## Abstract

**Background:**

The aim of this retrospective study was to identify high-risk dental extraction patients and the timing of postoperative hemorrhage to evaluate whether preventive hospitalization should be considered in patients on antiplatelet medication (AP) or anticoagulants.

**Methods:**

Our study included 1595 procedures; 1319 were conducted under monotherapy (Group I: AP; Group II: indirect oral anticoagulant [IAC]; Group III: direct oral anticoagulant [DOAC]) and 276 under dual therapy (Group IV: double AP; Group V: AP and IAC; Group VI: AP and DOAC). We evaluated the incidence, frequency and timing of hemorrhage, hospitalization rate, and treatment of bleeding incidents.

**Results:**

The incidence of hemorrhagic events was significantly higher in the dual therapy groups compared to the monotherapy groups (*p* < 0.001). Comparing the procedures under monotherapy, those on DOAC (Group III) had a significantly higher risk of postoperative bleeding than Groups I and II (*p* < 0.001) and a higher rate of repeated bleeding episodes (*p* = 0.035). Regarding bleeding incidents, 44% (dual therapy) vs. 51.1% (monotherapy) occurred on the day of surgery.

**Conclusions:**

The bleeding risk after dental extractions is overall low and patients were often hospitalized preventively due to their comorbidities rather than actual bleeding risk. Patients should be instructed about local compression, and surgeries should be completed in the morning to avoid emergency visits. However, patients with cardiovascular diseases and dual therapy had a higher risk of postoperative hemorrhage. Thus, hospitalization is to be considered in these cases.

**Trial registration:**

The study was approved by the Ethics Committee of the Medical Faculty of RWTH Aachen (Decision Number 24-136). This was a retrospective clinical study designed to analyze postoperative bleeding and hospitalization rates after dental extractions in patients on AP or anticoagulation therapy.

## Background

In an aging population, the growing number of patients on antiplatelet medication (AP) or anticoagulation therapy can be a challenge in oral and maxillofacial surgery, and anticipation of the risk of postoperative bleeding plays an important role in the preoperative planning of dental extractions [1–3].

As the German S3 guidelines for the management of anticoagulation and antiplatelet therapy in dental surgery recommends hospitalization of patients with extractions under continuous IAC and dual AP, preoperative hospitalization is still quite common in Germany [4]. This is an enormous cost factor and the decision for preventive hospitalization to monitor bleeding episodes is often based on the surgeon´s personal experience and evaluation [5] as there is a lack of evidence, if patients benefit from an inpatient treatment.

There is a whole range of blood-thinning medication: AP inhibits platelet aggregation, such as acetylsalicylic acid (ASA), clopidogrel and ticagrelor [6]. Indirect oral anticoagulants do not directly inhibit the clotting factors but mediate through antithrombin (low weight molecular heparin [LWMH]) or prevent the activation of the vitamin K-dependent clotting factors (phenprocoumon); direct anticoagulants are defined as anticoagulants that directly inhibit the clotting factors Xa or Thrombin [7].

Some anticoagulants may be associated with a higher bleeding risk than others, but the specific incidence of heavy and light postoperative hemorrhage and its temporary occurrence after the operation often remains unclear [8, 9].

While AP therapy with ASA does not seem to be associated with a higher risk of hemorrhage compared to healthy patients, dual AP therapy increases the risk for postoperative bleeding after tooth extraction [10]. A multicenter study by Hiroshi et al. addressing extractions in patients taking direct oral anticoagulants (DOACs) and warfarin showed no significant difference in postoperative bleeding events between these medications [11]. However, Miranda et al. found a significantly higher risk of hemorrhage after teeth extraction in patients who had undergone replacement of warfarin with LWMH compared to patients taking DOACs [12].

Growing evidence exists that the interruption of anticoagulants and bridging is neither necessary nor worth the risk of thromboembolic incidents since bleeding after minor oral surgery is usually not life-threatening [13–16].

The incidence of postoperative bleeding can be lowered by applying hemostatic agents such as gelatin sponges or collagen fleece [17, 18] or by the use of tranexamic mouthwash [19].

The variety of anticoagulant medications and their combinations must be set in context with the extent of dental surgery, which was analyzed in a retrospective study by Ueda et al., who found higher rates of postoperative bleeding in patients who had undergone osteotomy, vertical incisions, and posterior or multiple extractions [20].

Although numerous studies have evaluated the risk of postoperative hemorrhage, the exact timing of postoperative bleeding and the setting (i.e., inpatient or outpatient), which plays an important role in preoperative planning and management, were not included. In some patients, hospitalization is necessary, and emergency presentations can be a burden, especially for patients with comorbidities [21, 22].

Hence, a detailed risk assessment is important not only to give dentists and patients a basis for their decision, but also of serious economic interest because unnecessary hospitalization is a high cost for the healthcare system.

The aim of this retrospective study was to identify high-risk dental extraction patients and the timing of postoperative hemorrhage to evaluate whether preventive hospitalization should be considered in patients on AP medication or anticoagulants.

## Methods

### Study design

The study was approved by the local Clinical Research Ethics Committee (Decision Number 24–136).

This was a retrospective clinical study designed to analyze postoperative bleeding and hospitalization rates after dental extractions in patients on AP or anticoagulation therapy.

### Sample size calculation

The existing literature on clinical studies on postoperative bleeding after dental extractions on AP and anticoagulation therapy was reviewed to calculate a suitable range for the sample size and define the investigation period.

In particular, the required sample size was derived based on studies by Ueda [20] et al., who evaluated postoperative bleeding occurrence after dental extractions in older patients receiving anticoagulation therapy in 395 patients, and by Zirk et al., who analyzed hemorrhage in 741 extraction sites treated with a collagen fleece [17].

The incidence of postoperative bleeding was the primary outcome considered when calculating the sample size.

The statistical program G* Power Version 3.1.9.6 (Heinrich-Heine-Universität, Düsseldorf, Germany) was used for the calculation, with an alpha value of 0.05, an effect size of 0.1, and a statistical power of 90%. Based on these parameters, a sample size of at least 1073 procedures was found to test the null hypothesis that there is no significant difference concerning postoperative bleeding events, with 90% power and a 95% confidence interval.

### Data collection

Data were collected on patients who had a dental extraction under AP or anticoagulation therapy between 2014 and 2023 in our university hospital department.

The exclusion criteria were patients with incomplete postoperative documentation, patients who had other procedures done during the same operation (e.g., biopsies, root resections), and patients aged under 18. To obtain a comparable and homogenous study population, patients with triple anticoagulation therapy were also excluded.

Our final study sample included 1595 surgeries performed in 1280 patients. All procedures were divided into two main groups (monotherapy vs. dual therapy) and six subgroups. Patients taking a single AP medication, such as ASA and clopidogrel, were assigned to Group I. Patients on indirect anticoagulation (IAC) (low molecular weight heparin [LMWH] or warfarin) were assigned to Group II, and all patients with a single DOAC (dabigatran, rivaroxaban, apixaban, or edoxaban) were included to Group III. The patients on dual medications were further assigned to three subgroups: Group IV for patients taking two AP drugs (ASA in combination with clopidogrel, prasugrel or ticagrelor); Group V for patients taking an AP medication in combination with IAC (ASA in combination with LMWH or warfarin); and Group VI for patients who had a combined therapy regimen of an AP medication with a DOAC (Table 1).

**Table 1:** Patient collective and group distribution

Type of therapy	Groups	Type of medication	Number of procedures	Medication
Mono therapy	I	Single AP	442	ASA (398) Clopidogrel (44)
	II	Single IAC	479	Warfarin (306) LMWH (173)
	III	Single DOAC	398	Dabigatran (24) Rivaroxaban (148) Apixaban (170) Edoxaban (56)
Dual therapy	IV	Dual AP	105	ASA + clopidogrel (75) ASA + prasugrel (10) ASA + ticagrelor (20)
	V	AP + IAC	93	ASA + warfarin (53) ASA + LMWH (40)
	VI	AP + DOAC	78	ASA + DOAC (46) (2x dabigatran; 18x rivaroxaban; 15x apixaban; 11x edoxaban) Clopidogrel + DOAC (32) (16x rivaroxaban; 11x apixaban; 5x edoxaban)

Legend: AP: antiplatelet medication; ASA: acetylsalicylic acid; IAC: indirect anticoagulant; DOAC: direct oral anticoagulant; LMWH: low molecular weight heparin

A total of 5649 teeth were removed. Of these, most (93.4%) were removed by extraction (5280 in total; 4298 in patients on monotherapy and 982 in patients on dual therapy), and 6.5% had to be removed by osteotomy (369 in total; 303 in patients on monotherapy and 66 in patients on dual therapy).

Of the procedures, 200 included only dental extractions only in the anterior region, 810 procedures took place in the posterior region, and 585 procedures included both anterior and posterior tooth removal. All procedures included the use of a gelatin sponge (Gelastypt^®^, Sanofi-Aventis, Frankfurt am Main, Germany), which was applied to the alveolus after extraction and the suturing afterwards.

After collecting the data from the medical records, we evaluated the patients’ health status and the setting (i.e., inpatient or outpatient) at the time of the operation. Regarding comorbidities, 682 procedures involved patients with cardiovascular diseases, 205 with neurologic disorders, 212 with metabolic issues, and 158 with orthopedic disabilities. Of the procedures, 1317 operations were performed in an outpatient setting, and 278 were scheduled as preventive inpatient interventions.

We evaluated the incidence, frequency, and timing of postoperative hemorrhage, emergency hospitalization rate due to oral bleeding events, and treatment of the bleeding incidents.

### Statistical analysis

For the categorical data (sex, region of surgery, comorbidities, pausing of medication, procedure setting, incidence of postoperative bleeding, surgical treatment of hemorrhage, and emergency hospitalization), the differences between the groups were analyzed using a chi-square test or the Freeman–Halton test. Continuous data (age, number of teeth extracted, and timing and frequency of postoperative bleeding) were evaluated using the Kruskal–Wallis test or Mann–Whitney test, as they lacked a Gaussian distribution according to the Shapiro–Wilk test. The risk of postoperative bleeding was assessed by applying a logistic regression model and using sex, comorbidities, pausing of medication and hospitalization as explanatory variables. P values < 0.05 were considered significant.

## Results

Comparing the patient groups on monotherapy, there were statistically significant differences in sex (*p* < 0.001). The median ages of the patients on AP monotherapy (Group I), IAC monotherapy (Group II), and DOAC monotherapy (Group III) were 67 (IQR ± 15), 68 (IQR ± 22), and 76 (IQR ± 16) years, respectively, which was a significant difference between the groups (*p* < 0.001).

Most teeth could be removed by extraction (93.4%), and the median number of extractions and osteotomies did not differ between the three monotherapy groups. The region of surgery was distributed relatively homogenously, but there were significant differences in procedures in which both anterior and posterior teeth were removed in favor of Group III (*p* = 0.043). In addition, the distribution of comorbidities differed significantly, with the patients in Group III being more likely to suffer from cardiovascular, neurologic, metabolic, or orthopedic diseases (all *p* < 0.001). The patients taking only a single AP drug (Group I) paused their medication significantly less often compared to the other two groups (*p* < 0.001). The setting of the procedure also differed significantly, as patients from Group III were more often hospitalized compared to the other two monotherapy groups (*p* < 0.001) (Table 2).

**Table 2:** Group-based characteristics of procedures on monotherapy

	Antiplatelet monotherapy (Group I)		Indirect anticoagulants monotherapy (Group II)	Direct oral anticoagulants monotherapy (Group III)	Total	*p* value
**Number of procedures**						
		**442**	**479**	**398**	**1319**	
**Sex**						
Male	280 (63.3%)		329 (68.7%)	221 (55.5%)	830 (62.9%)	**< 0.001**
Female	162 (36.7%)		150 (31.3%)	177 (44.5%)	489 (37.1%)	
**Median age**						
	67 (IQR ± 15)		68 (IQR ± 22)	76 (IQR ± 16)	70 (IQR ± 19)	**< 0.001**
**Region of surgery**						
Anterior teeth only		52 (11.7%)	67 (14.0%)	42 (10.6%)	161 (12.2%)	0.285
Posterior teeth only		236 (53.4%)	252 (52.6%)	192 (48.2%)	680 (51.6%)	0.278
Both		154 (34.8%)	160 (33.4%)	164 (41.2%)	478 (36.2%)	**0.043**
**Median number of extracted teeth**						
Extractions		2 (IQR ± 3)	2 (IQR ± 3)	2 (IQR ± 4)	2 (IQR ± 3)	0.100
Osteotomies		0 (IQR ± 0)	0 (IQR ± 0)	0 (IQR ± 0)	0 (IQR ± 0)	0.862
**Comorbidities**						
Cardiovascular		76 (11.1%)	160 (33.4%)	263 (66.1%)	499 (37.8%)	**< 0.001**
Neurologic		29 (6.6%)	49 (10.2%)	68 (17.1%)	146 (11.1%)	**< 0.001**
Metabolic		33 (7.5%)	54 (11.3%)	73 (18.3%)	160 (12.1%)	**< 0.001**
Orthopedic		16 (10.1%)	33 (6.9%)	41 (10.3%)	90 (6.8%)	**< 0.001**
**Pausing of medication prior to the operation**						
Yes	4 (0.9%)		53 (11.1%)	267 (67.1%)	324 (24.6%)	**< 0.001**
No	438 (99.1%)		426 (88.9%)	131 (32.9%)	995 (75.4%)	
**Setting**						
Outpatient	401 (90.7%)		428 (89.4%)	304 (76.4%)	1133 (85.9%)	**< 0.001**
Inpatient	41 (9.3%)		51 (10.6%)	94 (23.6%)	186 (14.1%)	

Legend: For the categorical data (sex, region of surgery, comorbidities, pausing of medication, and procedure setting), the differences between the groups were analyzed using chi-square tests or the Freeman–Halton test. The continuous data (age and median number of extractions) were evaluated using the Kruskal–Wallis test, as they lacked a Gaussian distribution according to the Shapiro–Wilk test. P values < 0.05 were considered significant

Looking at the patient groups on dual medication, the age distribution differed significantly in favor of Group VI (*p* < 0.001), but sex did not (*p* = 0.632). Similar to the patients on monotherapy, there was a significant difference between the groups concerning teeth removal in both the anterior and posterior areas (*p* = 0.027). Most tooth removals were simple extractions without the need for osteotomy (93.7%), but there was a significant difference between the groups in the median number of teeth removed (*p* = 0.012 for extractions; *p* = 0.022 for osteotomies). The distribution of comorbidities did not differ significantly, except for neurologic diseases, which were present significantly more often in Group VI (*p* < 0.001). While pausing of medication was also significantly more often present in patients taking DOAC combined with an AP drug (*p* < 0.001), the setting of the operation did not differ between the three groups taking dual therapy (*p* = 0.955) (Table 3).

**Table 3:** Group-based characteristics of procedures on dual therapy

	Dual Antiplatelet therapy (Group IV)		Indirect anticoagulants combined with antiplatelet (Group V)	Direct oral anticoagulants combined with antiplatelet (Group VI)	Total	*p* value
**Number of procedures**						
		**105**	**93**	**78**	**276**	
**Sex**						
Male	74 (70.5%)		71 (76.3%)	56 (71.8%)	201 (72.8%)	0.632
Female	31 (29.5%)		22 (23.7%)	22 (28.2%)	75 (27.2%)	
**Median age**						
	63 (IQR ± 19)		72 (IQR ± 21)	73 (IQR ± 16)	69 (IQR ± 20)	**< 0.001**
**Region of surgery**						
Anterior teeth only		14 (13.3%)	15 (16.2%)	10 (12.8%)	39 (14.1%)	0.790
Posterior teeth only		55 (52.4%)	47 (50.5%)	28 (35.9%)	130 (47.1%)	0.063
Both		36 (34.3%)	31 (33.3%)	40 (51.3%)	107 (38.8%)	**0.027**
**Number of extracted teeth**						
Extractions		2 (IQR ± 3)	3 (IQR ± 4)	3 (IQR ± 5)	2 (IQR ± 4)	**0.012**
Osteotomies		0 (IQR ± 0)	0 (IQR ± 0)	0 (IQR ± 0)	0 (IQR ± 0)	**0.022**
**Comorbidities**						
Cardiovascular		71 (67.6%)	58 (62.4%)	54 (69.2%)	183 (66.3%)	0.599
Neurologic		21 (20%)	10 (10.8%)	28 (35.9%)	59 (21.4%)	**< 0.001**
Metabolic		21 (20%)	18 (19.4%)	13 (16.7%)	52 (18.8%)	0.840
Orthopedic		29 (27.6%)	17 (18.3%)	22 (28.2%)	68 (24.6%)	0.216
**Pausing of at least one drug prior to the operation**						
Yes	3 (2.9%)		3 (3.2%)	30 (38.5%)	36 (13%)	**< 0.001**
No	102 (97.1%)		90 (96.8%)	48 (61.5%)	995 (87%)	
**Setting**						
Outpatient	69 (65.7%)		63 (67.7%)	52 (66.7%)	184 (66.7%)	0.955
Inpatient	36 (34.3%)		30 (32.3%)	26 (33.3%)	92 (33.3%)	

Legend: For the categorical data (sex, region of surgery, comorbidities, pausing of medication, and procedure setting), the differences between the groups were analyzed using chi-square tests or the Freeman–Halton test. The continuous data (age, median number of extractions) were evaluated using the Kruskal–Wallis test, as they lacked a Gaussian distribution according to the Shapiro–Wilk test. P values < 0.05 were considered significant

**Fig. 1:**
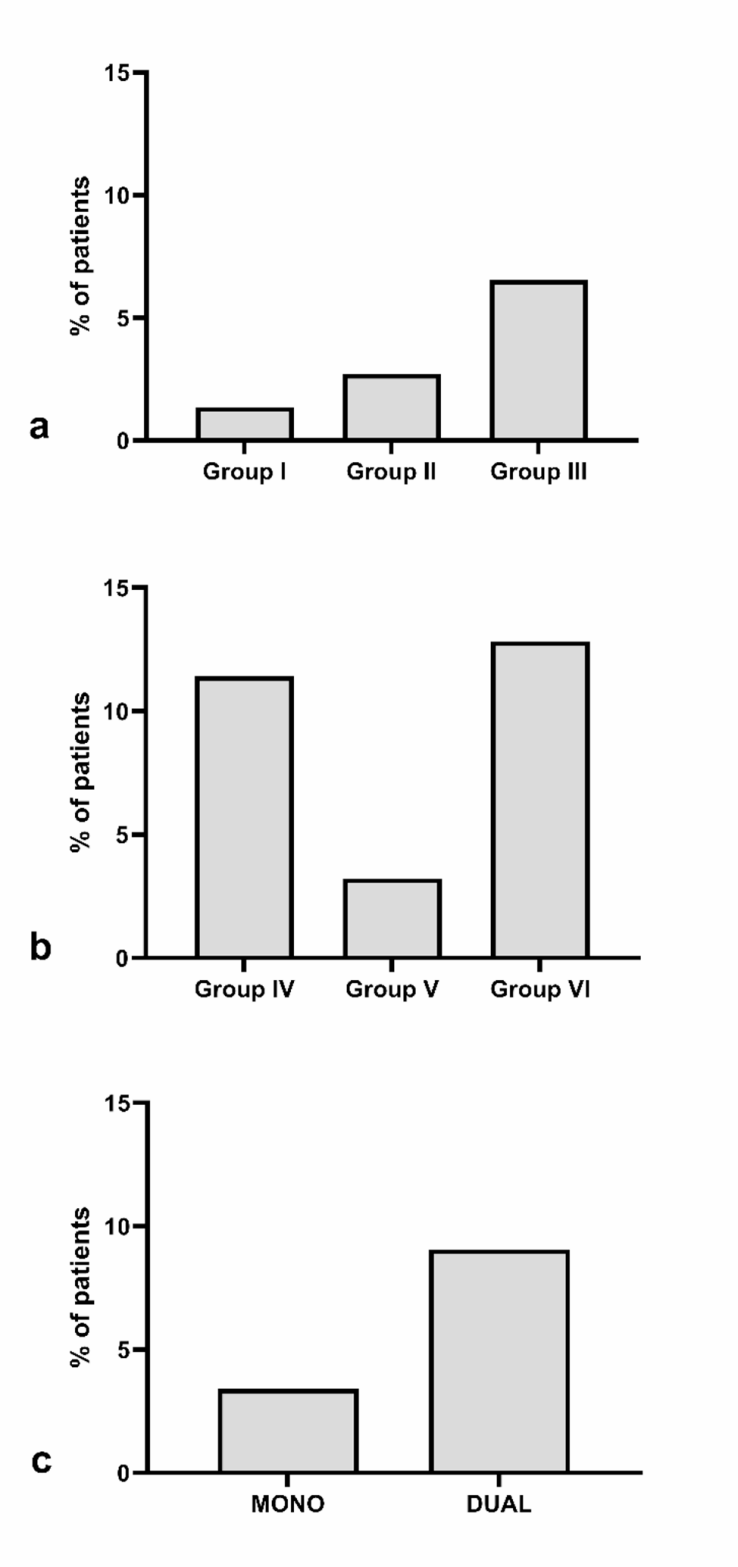
Comparison of postoperative bleeding events between the groups ***(a)**** In the 1319 procedures of patients on monotherapy*,* 45 postoperative bleeding episodes were identified*,* and there was a significant difference between the groups in favor of Group III (p < 0.001).****(b)**** In the 276 procedures involving dual therapy*,* 25 postoperative bleeding events occurred. No significant difference was found between Groups IV*,* V*,* and VI (p = 0.052).****(c)**** The incidence of hemorrhagic events was significantly more frequent than in the groups on dual therapy compared to those on monotherapy (p < 0.001)*

**Fig. 2:**
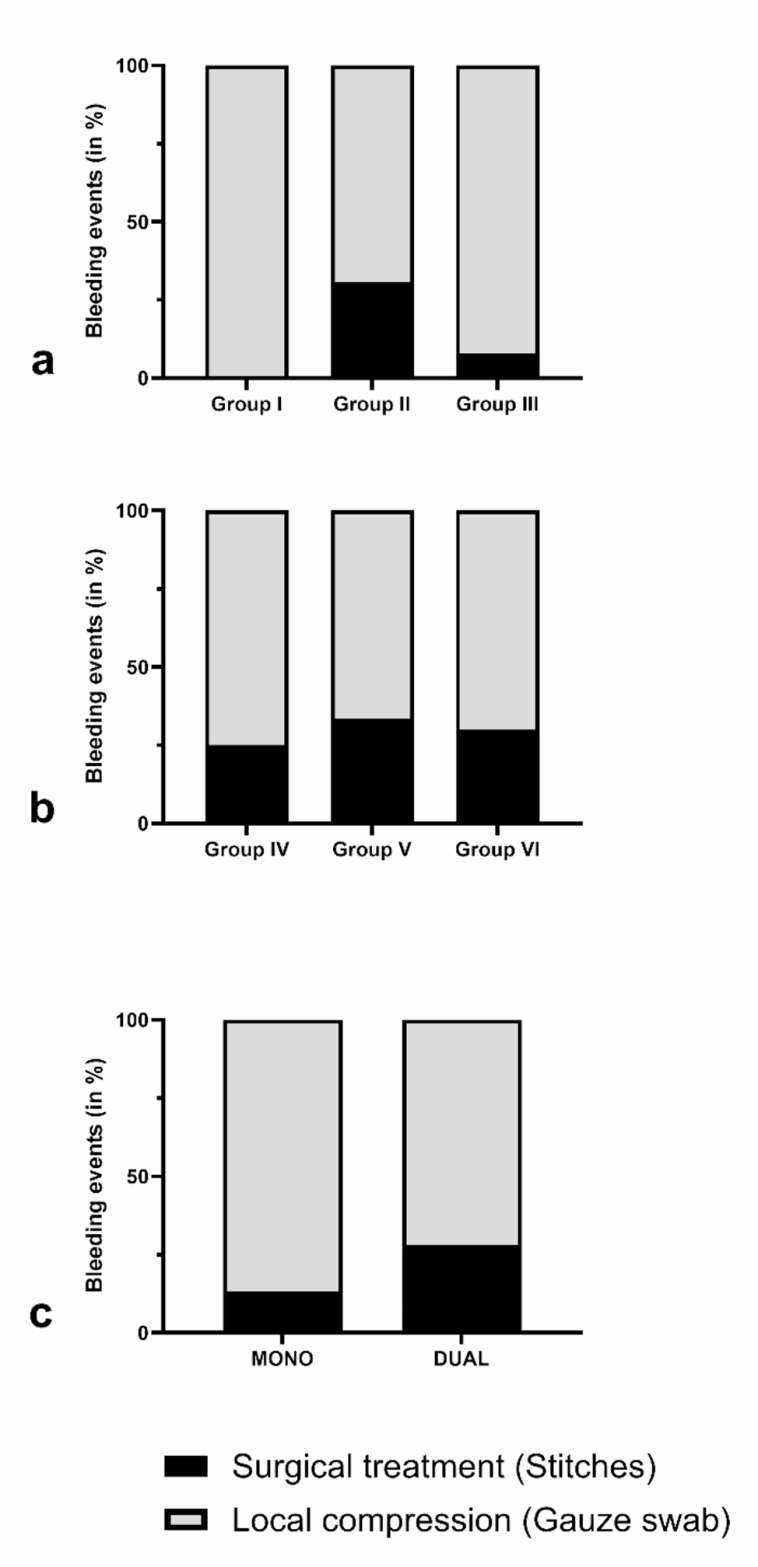
Comparison of the treatment of bleeding events between the groups ***(a)**** Therapy of bleeding episodes (local compression vs. stitching) differed neither between the Groups I–III (p = 0.142)*, *nor****(b)**** between Groups IV–VI (p = 0.100)*, ***(c)**** nor between monotherapy compared to dual therapy*. *(p = 0.131)*

In the 1319 procedures of patients taking monotherapy, 45 postoperative bleeding episodes were identified (3.4%), of which 6 were in patients on AP medication, 13 on IAC and 26 on DOAC, which was a significant difference in favor of the patients taking DOAC (Group III) (*p* < 0.001) (Fig. 1a). Of these hemorrhage events, 39 could be solved with local compression, but 6 needed additional stitching (4 in Group II and 2 in Group III), which was not significantly different between the groups (Fig. 2a). In 24 of these events, the bleeding episode happened in an inpatient setting (Group I: 3; Group II: 8; Group III: 13; *p* = 0.912). In the 21 events that occurred in an outpatient setting, 3 patients had to be hospitalized to be monitored afterwards, which was not statistically significant (Group II: 2; Group III: 1; *p* = 0.519).

Of the bleeding events, 23 took place on the day of the operation (51.1%), and 93.3% of all bleeding events occurred up to the third postoperative day (Table 4). No significant difference was found between the median day of hemorrhage (*p* = 0.714) between the Groups I, II, and III, but there was a significant difference in the number of repeated bleeding events comparing the three groups on monotherapy in favor of the group undergoing procedures on DOAC (*p* = 0.035).

In the 276 procedures involving dual therapy, 25 postoperative bleeding events occurred. Of these, 12 were in patients on double AP medication, 3 were on AP combined with IAC therapy, and 10 were taking an AP and a DOAC, and no significant difference was found between the Groups IV, V, and VI (*p* = 0.052) (Fig. 1b). Of these events, 7 were treated surgically by stitching (Group IV: 3; Group V: 1; Group VI: 3; *p* = 1.00) (Fig. 2b).

Of these events, 14 happened in an inpatient setting (*p* = 0.866), and no patient had to be hospitalized because of a bleeding episode. Of the hemorrhages, 44% happened on the same day of the procedure, and 80% of all bleeding events occurred during the first two days after the operation (Table 4). No significant difference was found between the median day of hemorrhage (*p* = 0.127) or the number of repeated hemorrhagic events (*p* = 0.796) between the Groups IV, V, and VI.

**Table 4:** Time of postoperative hemorrhage comparing between monotherapy and dual therapy

Time	Monotherapy		Dual therapy	
	Events	Cumulative percentage	Events	Cumulative percentage
**Day of the surgery**	23 (51.1%)	51.1%	11 (44%)	44%
**1st day**	9 (20%)	71.1%	6 (24%)	68%
**2nd day**	4 (8.9%)	80%	3 (12%)	80%
**3rd day**	6 (13.3%)	93.3%	2 (8%)	88%
**4th day**	-	93.3%	2 (8%)	96%
**5th day**	1 (2.2%)	95.6%	1 (4%)	100%
**7th day**	1 (2.2%)	97.8	-	100%
**10th day**	1 (2.2%)	100%	-	100%
**Total**	45	100%	25	100%

The incidence of hemorrhagic events was significantly greater in the groups on dual therapy compared to those on monotherapy (*p* < 0.001) (Fig. 1c).

No significant difference was found between the number of extractions and osteotomies comparing the patients with and without postoperative bleeding (*p* = 0.230 for extractions in procedures with monotherapy; *p* = 0.226 for osteotomies in procedures with monotherapy; *p* = 0.102 for extractions in procedures with dual therapy; *p* = 0.736 for osteotomies in procedures with dual therapy).

Regarding the bleeding episodes in the monotherapy and dual therapy groups, the treatment (local compression vs. stitching) did not differ significantly between the groups (*p* = 0.142 for Groups I–III; *p* = 1.00 for Groups IV–VI; *p* = 0.131 for monotherapy compared to dual therapy) (Fig. 2c).

Neither the number of repeated bleeding events (*p* = 0.859), nor the inpatient or outpatient setting (*p* = 0.830), nor postoperative hospitalization (*p* = 0.187) differ significantly between the patients on monotherapy or dual therapy.

However, both in the patients taking monotherapy as well as in the patients on dual therapy, pausing of medication led to significantly higher chances of postoperative bleeding episodes (*p* < 0.001 for monotherapy; *p* = 0.088 for dual therapy).

While 33 bleeding episodes happened in an inpatient setting, 245 procedures were carried out under preventive hospitalization, and these patients did not suffer from hemorrhage afterwards. Compared to the patients treated in an outpatient setting, they suffered significantly more often from cardiovascular, neurologic, metabolic, and orthopedic diseases (all *p* < 0.001).

Furthermore, we performed a regression analysis in order to control for patient specific variables: When analyzing the risk for postoperative bleeding between patients on mono and dual therapy and adjusting for sex, comorbidities, pausing of medication and hospitalization, bleeding was significant more often in patients with dual medications (*p* = 0.005), patients with cardiovascular disease (*p* = 0.004), patients who paused their medication (*p* = 0.002) and patients who were hospitalized preventively (*p* < 0.001) (Table 5).

**Table 5:** Logistic regression analysis performed for evaluation of postoperative bleeding

Parameter	Comparison	Odds Ratio	*p* value
Medication regimen	Mono vs. Dual therapy	2.315 (CI 1.290–4.149)	**0.005**
Sex	Male vs. Female	0.715 (CI 0.410–1.246)	0.237
Cardiovascular diseases	No vs. Yes	2.690 (CI 1.379–5.248)	**0.004**
Neurologic diseases	No vs. Yes	1.321 (CI 0.740–2.359)	0.347
Metabolic diseases	No vs. Yes	1.613 (CI 0.921–2.825)	0.095
Orthopedic diseases	No vs. Yes	1.640 (CI 0.883–3.048)	0.118
Pausing of medication	No vs. Yes	2.397 (CI 1.362–4.219)	**0.002**
Hospitalization	No vs. Yes	2.574 (CI 1.543–4.295)	**< 0.001**

Legend: CI: confidence interval

When controlling for sex, comorbidities (cardiovascular, neurologic, metabolic, and orthopaedic), pausing of medication, and hospitalization, postoperative bleeding was significant more often in patients with dual medications (*p* = 0.005), patients with cardiovascular disease (*p* = 0.004), patients who paused their medication (*p* = 0.002) and patients who were hospitalized preventively (*p* < 0.001)

## Discussion

Numerous studies have evaluated bleeding after dental extractions under continuous or discontinuous medication with AP, IAC, or DOAC, but the circumstances, exact timing, and hemorrhage extent are often not addressed [20, 23]. While medication and monotherapy or dual therapy regimens play an important role, the use of hemostatic materials and wound suturing can prevent most postoperative bleedings [17, 18]. However, risk assessment is an important factor for patient comfort and for reducing costs [4, 21, 22, 24, 25]. The present study divided all procedures depending on which medication they were carried out under into two main groups and six subgroups, which made it possible to compare not only single drugs but also common dual combinations.

To the best of our knowledge, this study was the first to evaluate the frequency, timing, and extent of postoperative bleeding after tooth extraction over a 10-year period in a large sample size of over 1500 procedures and 5649 teeth removals.

Since postoperative bleeding after dental extractions is a relatively rare event, with an incidence of 0.4–8.6% overall [8, 13, 26], it is important to look at a large study population, which has only been done in very few trials before [8, 27].

In our study, the incidence of postoperative hemorrhage was 3.4% after procedures involving single AP or anticoagulation therapy (1.3% for AP, 2.7% for IAC, and 6.5% for DOAC), which was a slightly higher rate for DOAC and a lower rate for IAC compared to a trial by Hiroshi et al., who reported 1.65–3.41% in patients on DOAC and 3.63% in patients on warfarin [11]. Unfortunately, the INR value was not documented for all the procedures under phenprocoumon, which is a limitation of our study concerning comparability of the procedures under IAC.

In this analysis, the patients on single AP or anticoagulation therapy had less postoperative bleeding events than the patients on dual therapy. This is in line with a study by Girotra et al. [28] and in contrast to the study by Lu et al. [29], while it has to be noted that both studies included a smaller number of procedures and only investigated double AP therapy. This study is one of the first to not only evaluate the risk for hemorrhage under dual AP therapy, but also to address the risk of the combined therapy of AP and IAC and AP combined with DOAC.

Bleeding rates after dental extractions in patients receiving DOAC therapy differ: while Hiroshi et al. and Hua et al. found lower rates compared to procedures under warfarin [9, 11], Ono et al. described comparable percentages of bleeding events in DOAC and vitamin K antagonists [27]. In a study by Inokoshi et al. [30], as well as in this 

study, the procedures under DOAC therapy had significantly more and more frequent postoperative bleeding episodes than oral surgery under IAC.

While interruption of AP and anticoagulation seems to be unnecessary [29, 31, 32] and an avoidable risk for thromboembolic incidents [14, 33, 34], we found a significantly higher risk for postoperative hemorrhage after procedures for which medication was paused beforehand. Since interruption of medication is usually only done for 24–48 h before the operation, this might just delay the occurrence of bleeding: 48.9% of all postoperative bleeding episodes under monotherapy occurred from the 1st to the 10th day after the operation.

Another explanation might be that the surgeon or patient feels misleadingly safe by interrupting the medication and hence not applying the same precautionary measurements or following postoperative instructions, such as local compression of the wound or soft nutrition.

A retrospective study by Ueda et al. found more bleeding episodes in patients who underwent vertical incisions, osteotomies, and posterior or multiple tooth extractions. In our study, there was no significant difference between the number of extractions and osteotomies between the procedures with and without postoperative bleeding. However, we did not include the criteria of mucosal or periosteal incision, which might have influenced bleeding events.

It is known that collagen, gelatin, and other hemostatic agents are effective in minimizing bleeding after oral surgery [17, 18, 21]. Postoperative instructions seem to be crucial to prevent emergency presentations due to postoperative bleeding [35]. In our study, as well as in multiple other studies, most bleeding episodes could be controlled by administration of local compression only [22, 24, 32, 36, 37], so it makes sense not only to educate the patient, but also to hand them a tranexamic mouth wash and gauze swabs for a possible bleeding event [38].

As described by Mauprivez et al., most bleeding incidents take place on the day of surgery [39], and 80% of all bleeding events in our study occurred during the first two days afterwards. This indicates, as suggested by Scully et al. [40], that oral surgery in patients with AP or anticoagulant therapy should be carried out in the morning and ideally early in the week to avoid emergency presentations outside of consultation hours.

Looking at the procedure setting, we found no significant difference between bleeding episodes that took place in an outpatient or in a preventive inpatient setting, nor did the emergency hospitalization rates differ significantly. This implies that preventive hospitalization did not cover those patients who were affected by bleeding. Although the German S3 guidelines for the management of anticoagulation and antiplatelet therapy in dental surgery [4] recommend hospitalization of patients with extractions under continuous IAC and dual AP, according to our study, there is a high share of patients who either do not bleed at all or bleed on the same day of the surgery, so that most patients would not profit from a preventive hospitalization overnight. This finding is in line with the study by Buchbender et al. and Eichhorn et al., who also described a very low incidence of emergency hospitalization due to postoperative bleeding [21, 36].

Nevertheless, it must be stated that the patients who were hospitalized preventively and did not bleed at all were significantly more likely to suffer from cardiovascular, neurologic, metabolic, and orthopedic diseases. Hence, the decision of the physician to monitor them postoperatively might be more likely to be associated with their general health and social factors than their actual bleeding risk. Still, especially patients under dual therapy and patients with cardiovascular disease were more likely to be affected by postoperative bleeding episodes and could benefit from postoperative observation.

## Conclusions

The risk of bleeding after dental extractions is low overall, but patients on dual therapy, have a higher risk compared to other monotherapy regimens. Most bleeding episodes took place on the day of the surgery, could be solved by local compression, and did not require surgical intervention. Hence, hospitalization is in most cases not necessary based on the incidence and temporary occurrence of bleeding episodes. In this study population, most patients were hospitalized preventively due to their comorbidities rather than their actual bleeding risk. However, patients with cardiovascular diseases and dual therapy had a higher risk of postoperative hemorrhage. Thus, hospitalization is to be considered in these cases. Further prospective studies analyzing the risk of postoperative bleeding and the necessity for hospitalization of high-risk patients should be performed.

## Data Availability

Availability of data and materials: The datasets used and/or analyzed during the current study available from the corresponding author on reasonable request.

## List of Abbreviations

AP: Antiplatelet
ASA: Acetylsalicylic acid
DOAC: Direct oral anticoagulant
LWMH: Low molecular weight heparin
IAC: Indirect anticoagulation
IQR: Interquartile range
CI: Confidence interval
